# Multispectral Optoacoustic Tomography Enables In Vivo Anatomical and Functional Assessment of Human Tendons

**DOI:** 10.1002/advs.202308336

**Published:** 2024-03-06

**Authors:** Ivana Ivankovic, Hsiao‐Chun Amy Lin, Ali Özbek, Ana Orive, Xosé Luís Deán‐Ben, Daniel Razansky

**Affiliations:** ^1^ Faculty of Medicine Institute for Biomedical Engineering and Institute of Pharmacology and Toxicology University of Zurich Winterthurerstrasse 190 Zurich CH‐8057 Switzerland; ^2^ Department of Information Technology and Electrical Engineering Institute for Biomedical Engineering ETH Zurich, Wolfgang‐Pauli‐Str. 27 Zurich CH‐8093 Switzerland; ^3^ Department of Biomedical Engineering and Environmental Sciences National Tsing Hua University No.101, Sec.2, Kuang‐Fu Rd Hsinchu 300044 Taiwan

**Keywords:** human tendon imaging, multispectral optoacoustic tomography, noninvasive imaging, tendon function, tendon vascularity

## Abstract

Tendon injuries resulting from accidents and aging are increasing globally. However, key tendon functional parameters such as microvascularity and oxygen perfusion remain inaccessible via the currently available clinical diagnostic tools, resulting in disagreements on optimal treatment options. Here, a new noninvasive method for anatomical and functional characterization of human tendons based on multispectral optoacoustic tomography (MSOT) is reported. Healthy subjects are investigated using a hand‐held scanner delivering real‐time volumetric images. Tendons in the wrist, ankle, and lower leg are imaged in the near‐infrared optical spectrum to utilize endogenous contrast from Type I collagen. Morphology of the flexor carpi ulnaris, carpi radialis, palmaris longus, and Achilles tendons are reconstructed in full. The functional roles of the flexor digitorium longus, hallicus longus, and the tibialis posterior tendons have been visualized by dynamic tracking during toe extension‐flexion motion. Furthermore, major vessels and microvasculature near the Achilles tendon are localized, and the global increase in oxygen saturation in response to targeted exercise is confirmed by perfusion studies. MSOT is shown to be a versatile tool capable of anatomical and functional tendon assessments. Future studies including abnormal subjects can validate the method as a viable noninvasive clinical tool for tendinopathy management and healing monitoring.

## Graphical abstract

Hand‐held multispectral optoacoustic tomography imaging of healthy tendons in human subjects demonstrates the potential for noninvasive, label‐free anatomical visualization and functional motion tracking. Furthermore, various vasculature groups in and around the Achilles tendon can be localized. Blood perfusion studies reveal a diversity of oxygenation dynamics in response to targeted exercise, providing insights into the contentious topic of tendon healing.

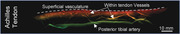

## 1 Introduction

Tendon pathologies (tendinopathy) are experienced by a vast proportion of the global population, representing the most frequent musculoskeletal disorders requiring medical attention.^[^ ^1^ ^]^ Tendon injuries are often painful and incapacitating and are commonly a result of sports injury, occupational overuse, or aging.^[^ ^2^ ^]^ While the Achilles tendon is the most frequently ruptured,^[^ ^3^ ^]^ the wrist flexor and foot tendons are also prone to injury. An injured tendon, primarily treated via physiotherapy, can undergo structural changes that directly impact its function of transmitting force from muscle to bone.^[^ ^2^ ^]^ Additionally, it is generally accepted that tendons have limited regeneration capacity due to sparse vascular supply.^[^ ^4^ ^]^ However, tendon vascularity is currently poorly understood, exemplified by a number of contradicting studies on perfusion response to exercise.^[^ ^5^, ^6^, ^7^, ^8^ ^]^ Diagnosis is typically based on physical examination,^[^ ^1^ ^]^ which is effective in locating site‐of‐pain and detecting structural abnormalities. Yet smaller‐scaled anatomical changes are often missed, along with vascular information. Thus, by developing new imaging methods to enable the simultaneous evaluation of microstructural deformation, blood perfusion, and oxygenation dynamics, tendon healing can be better understood and recovery treatments further improved.

Currently, magnetic resonance imaging (MRI), pulse‐echo and Doppler ultrasound (US) are the gold standards for clinical tendon imaging. MRI provides excellent anatomical information,^[^ ^9^ ^]^ nonetheless its low temporal resolution hinders direct functional assessment. Dynamic contrast‐enhanced MRI and US can be used to analyze the microcirculation of tendons after injuries, though exogenous contrast agents are required.^[^ ^10^ ^]^ US offers complementary information on real‐time motion and blood flow estimation but with limited diagnostic accuracy. As most US hand‐held scanners are cross‐sectional (2D), sensitivity to the plane‐of‐scanning leads to this approach being vulnerable to inter‐user variability.^[^ ^11^ ^]^

By capitalizing on rich endogenous contrast, multispectral optoacoustic tomography (MSOT) offers a versatile platform for fast (real‐time), volumetric (3D) rendering of molecular information in soft tissues up to a depth of several centimeters.^[^ ^12^ ^]^ Being a noninvasive label‐free approach, several clinical MSOT applications have already been demonstrated, e.g. for imaging of carotid artery morphology and function,^[^ ^13^, ^14^ ^]^ muscular dystrophy,^[^ ^15^ ^]^ or Crohn's disease.^[^ ^16^ ^]^ MSOT imaging of tendon anatomy has been performed on in vivo murine models,^[^ ^17^ ^]^ ex vivo swine samples,^[^ ^18^ ^]^ and arthritic finger joints.^[^ ^19^ ^]^ The collagen spectrum has also been reported for human tendons.^[^ ^16^ ^]^ Hyperemia in joints and the Achilles tendon have further been quantified with light‐emitting‐diode (LED) based systems.^[^ ^20^, ^21^ ^]^ Furthermore, tendon tissue anisotropy has been characterized ex vivo using fiber‐based polarized techniques.^[^ ^22^ ^]^ Nevertheless, to date, in vivo MSOT characterization of human tendons has yet to be explored. In this work, we investigate the feasibility of clinical MSOT assessment for tendon morphology and physiological function in response to exercise.

## 2 Result

### 2.1 Morphological Quantifications of Wrist Flexors

**Figure 1:**
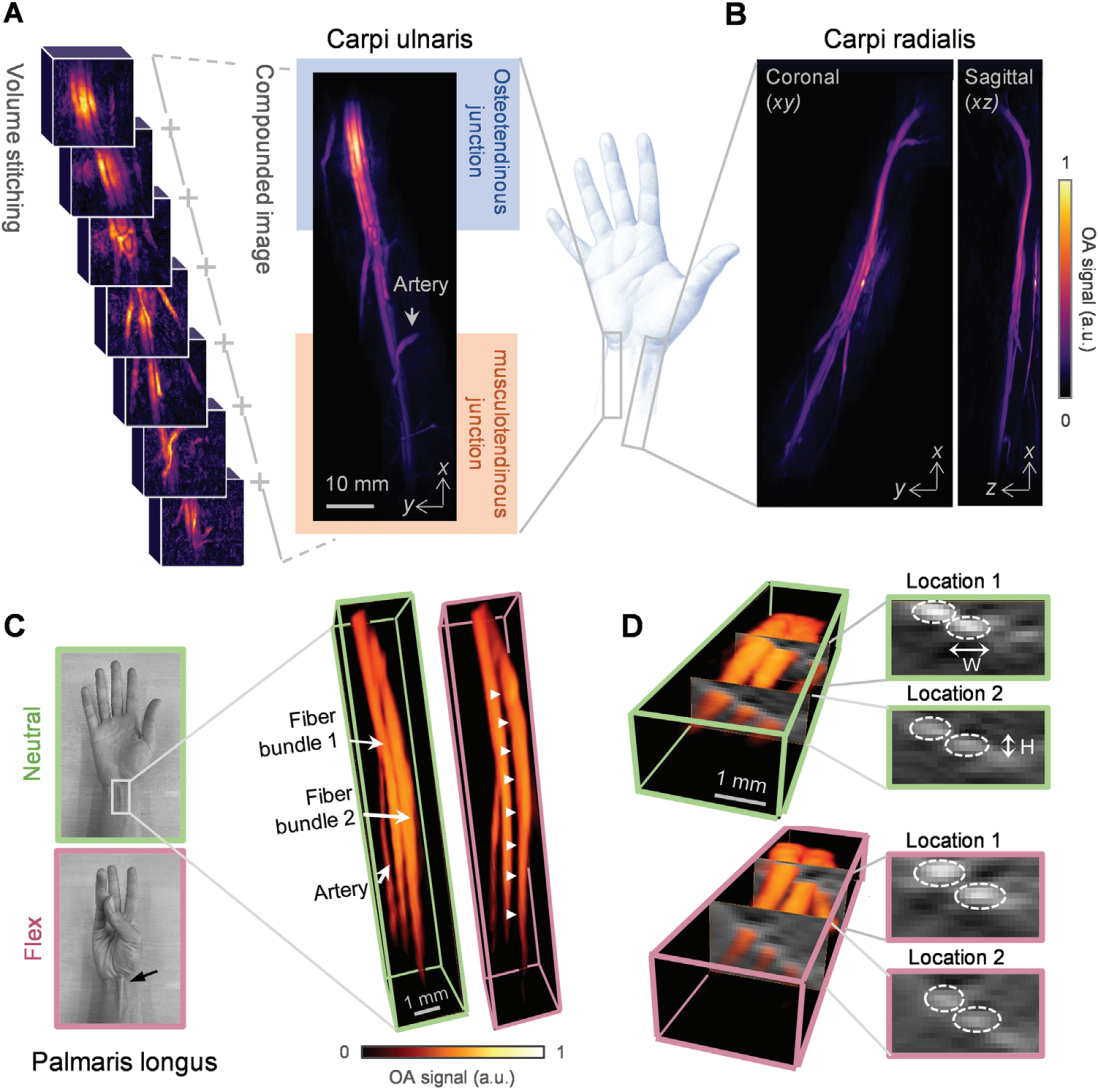
MSOT anatomical characterization of three wrist tendons. A) Carpi ulnaris tendon: (left) a sequence of frames for volumetric image stitching; and (right) large‐volume compounded image, featuring insertions into bone (osteotendinous junction, blue) and muscle (musculotendinous junction, orange). B) Carpi radialis tendon presented in the coronal (*xy*) and sagittal (*xz*) projections. C) Palmaris longus tendon: (left) photographs picturing the hand in the neutral, open‐hand state (green) and the flexed, test state (pink). The latter reveals the presence of the palmaris longus tendon (arrow), and (right) compounded volumes of the neutral and flexed states respectively. Individual fiber bundles (one and two) are depicted, along with nearby vasculature. D) Compounded volumes showing cross‐sections in two locations. The cross‐sections of both fiber bundles (one and two) are circled, allowing for width (W) and height (H) measurements.

We investigated three wrist flexor tendons: carpi ulnaris, carpi radialis, and palmaris longus; all elongated structures spanning from the wrist toward the middle of the forearm. Large‐volume compound images were reconstructed by acquiring and stitching together a series of continuous frames, pictured in **Figure** **1** (panel a). The projection of the carpi ulnaris in full (right) displays the osteotendinous junction at the wrist with its insertion into the ulnar muscle at the musculotendinous junction. An artery (labeled) could be seen pulsating during real‐time scanning (Video S1, Supporting Information). Figure 1b presents the carpi radialis in both coronal (*xy*) and sagittal (*xz*) projections, where the latter provides depth information, featuring the insertion into the radial muscle. Further, to test the feasibility of functional morphology characterization by MSOT, neutral (green) and flexed (pink) wrist states of both carpi ulnaris and carpi radialis tendons were contrasted, and their differences were highlighted in the overlay (Figure S1, Supporting Information). Next, to explore quantitative approaches for morphological analyses, the palmaris longus was examined. Figure 1c (left) pictures the neutral, open hand (green) juxtaposed to flexed (pink), the latter being the typical clinical presence‐identification test of the tendon (indicated) as it is absent in ≈14% of the population. The palmaris longus at both positions are presented in Figure 1c (right), where the individual fiber bundles could be distinguished (one and two) along with a neighboring artery (labeled). Structural differences between the two fibers were compared analytically, and an average displacement of 0.156 ± 0.025 mm was measured. Cross‐sectional images also facilitated the fiber dimension quantifications, depicted in Figure 1d, where the width (W) and height (H) of both bundles (circled) are summarized in Figure S2 (Supporting Information).

### 2.2 Real‐Time Flexion‐Extension Dynamics

**Figure 2:**
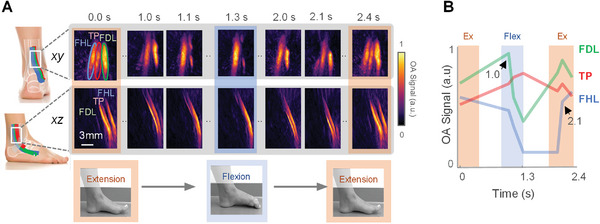
Real‐time motion tracking of three tendons simultaneously. A) The flexor digitorium longus (FDL), flexor hallicus longus (FHL), and tibialis posterior (TP) tendons are shown in *xy* and *xz* projections from 0 to 2.4 s between extension (orange) and flexion (blue) of the toes. B) Temporal MSOT intensity profiles extracted from each region of interest, confirming tendon function correlating to movement. As the hallux (big toe) initializes ankel plantarflexion, the FHL (governing tendon) signal decreases instantly, followed by a decrease in the FDL (governing the motion of the other four digits) signal starting at 1.0 s. TP signal remains relatively stable, where intensity drifts are attributed to FHL and FDL modulations.

Featured in **Figure** **2**, the flexor hallicus longus (FHL), flexor digitorium longus (FDL), and tibialis posterior (TP) tendons were tracked simultaneously during ankle movements alternating between extension (orange) and plantarflexion (blue). Figure 2a shows MSOT projections in the sagittal (*xy*) and coronal (*xz*) planes at selected instances (full video: Video S2, Supporting Information). Temporal intensity profiles were extracted from regions of interest of each tendon (panel b), leading to the observation that both FHL and FDL dynamics correlate closely to the plantarflexion‐extension motion: FHL signal decreases instantly, followed by a decrease in FDL signal starting at 1.0 s. As FHL and FDL govern digit movements of the hallux (big toe) and the four others respectively while TP controls whole foot motion, the results are congruent with the observation that the big toe initializes ankle plantarflexion. TP signal remains relatively stable, where intensity drifts can be attributed to FHL and FDL modulations.

### 2.3 Molecular Analysis of Achilles Tendon and Vasculature

**Figure 3:**
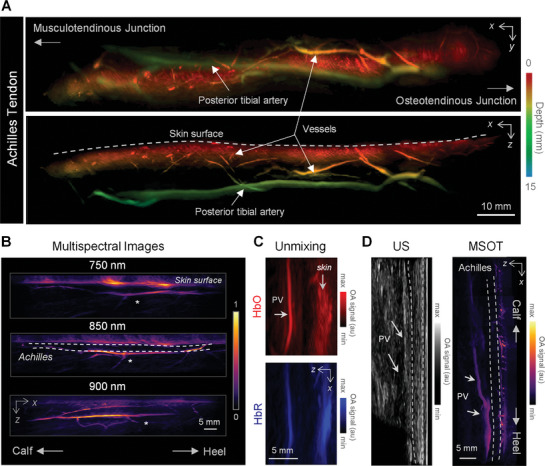
Characterization of the Achilles tendon. A) Volumetric MSOT image of the Achilles tendon shown projected in xy and xz planes. Depth was colour‐coded where red signifies the most superficial structures and blue the deeper structures. The posterior tibial artery and vessels surrounding the Achilles are labeled. B) MSOT scans of an Achilles tendon at 750, 850, and 900 nm wavelengths from the heel to the calf, where * depicts the same anatomical location. Deeper vessels become more visible for the longer wavelengths. C) Section of the Achilles tendon spectrally unmixed for bio‐distribution of oxygenated (HbO) and deoxygenated (HbR) hemoglobin. Both components are present in the PV. D) B‐mode ultrasound and MSOT images of the Achilles tendon of the same volunteer for anatomical validation. The Achilles tendon and the posterior vessel are both indicated.

A scan of the Achilles, from the calcaneus (osteotendinous junction) to the calf muscle insertion (musculotendinous junction), is presented as sagittal (*xy*) and coronal (*xz*) MIPs in **Figure** **3**, (panel a). The large‐volume reconstruction spans across a length of 13 cm, featuring three distinct types of surrounding vasculature: the prominent posterior tibial artery (the main blood supplier to the Achilles tendon), within‐tendon vessels, and superficial vessels. Depth

information is color‐coded to differentiate superficial structures (skin and superficial vessels – red) from deeper vessels (posterior – green/blue). Multispectral scans (750, 850, and 900 nm) further emphasized spectral features (panel b). Strong MSOT skin signal is present in the 750 nm image due to melanin absorption, whilst HbO absorption brings forth more visibility of deeper vessels at 850 and 900 nm. For a quantitative estimation of surrounding vasculature oxygen saturation, multispectral MSOT data (750, 780, 800, 850, 900, and 930 nm) of the Achilles at rest was acquired at ≈5 cm from the calcaneus. Bio‐distributions of oxygenated (HbO) and deoxygenated (HbR) hemoglobin were thereafter spectrally unmixed (Figure 3c). Both hemodynamic components are present in the large posterior vessel to the Achilles, though higher HbR concentrations were found within the skin. Side‐by‐side comparisons with B‐mode US cross‐sections serve as anatomical validations (Figure 3d).

### 2.4 Perfusion Response to Exercise

**Figure 4:**
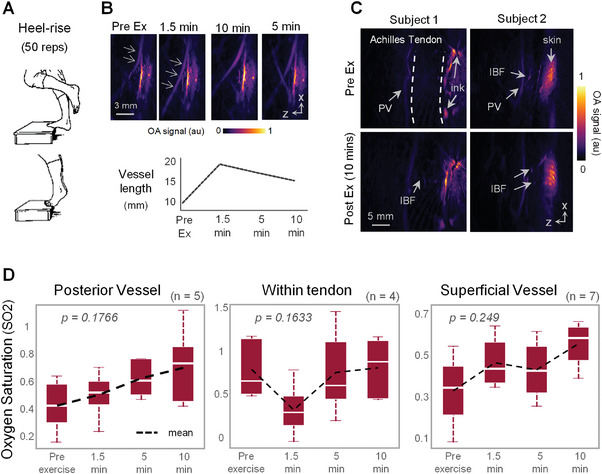
Perfusion analysis of the Achilles tendon in response to exercise. A) Schematic of exercise undertaken by volunteers involving 50 heel rises. B) Evaluation of vascular changes (indicated) in response to exercises. An increase in vessel length (mm) was recorded 1.5 min after exercise, followed by a decline for 5 and 10 min. C) MSOT projections of Achilles tendon vasculature from two subjects imaged pre‐ and post‐exercise (10 min), showing different intratendinous blood flow (IBF) dynamics. The skin was marked with ink for probe positioning. D) Quantification of oxygen saturation in response to exercise from three vascular regions near the Achilles tendon: posterior vessel, within tendon, and superficial vessel.

To gain more insights into vascular perfusion responses to exercise, eight healthy volunteers were asked to do 50 repetitions of Achilles‐targeting heel‐rises (**Figure** **4**, panel a). Each Achilles was imaged multispectrally at four time points: before exercise, and at 1.5, 5, and 10 min after. As shown in Figure 4b, changes in MSOT signals can be observed from as early as 1.5 min postexercise and remain evident throughout the 10 min duration. Selected vessel lengths (arrows) were measured at each time point. A minimum increase of 10.5 mm was observed at 1.5 min postexercise, evident in blood distribution changes. Intratendinous blood flow (IBF), a parameter associated with tendinopathy^[^ ^5^ ^]^ or a physiological exercise response,^[^ ^23^ ^]^ was detected in four subjects. Figure 4c demonstrates two examples: IBF in subject one becomes detectable post‐exercise, whereas in another (subject 2) where IBF is present also beforehand. Figure S3 (Supporting Information) (panel a) details MSOT dynamics of the three vascular regions (posterior, within the tendon, and superficial) at individual wavelengths (760, 800, 860, and 900 nm) from all subjects examined. Signal intensity changes reflect rapid physiological hemodynamics and showcases MSOT's ability to capture wide‐varying exercise responses. HbO and HbR components were then unmixed and normalized with their pre‐exercise values (panel b), leading to oxygen saturation estimations presented in Figure 4d. While no statistical significance was observed (posterior vessel, *p = 0.1766*, within the tendon, *p = 0.1633*, superficial vessel, *p = 0.249;* ANOVA), likely due to the low sample number, oxygen saturation in the posterior and superficial vessels tend to increase – an expected observation as blood flow to the calf muscle increases during exercise.^[^ ^24^ ^]^ Within‐tendon signals appear to drop 1.5 min after exercise, followed by a steady return to baseline. The observation of decreased IBF in conjunction with the increasing posterior vessel signal is in congruence with a Doppler US study recently reported,^[^ ^8^ ^]^ and could be ascribed to functional stunting, a phenomenon occurring when oxygen supply is directed to areas of high metabolic demand (calf muscle), whereas less active areas (Achilles tendon) experience a decrease in oxygen saturation.^[^ ^25^ ^]^ The agreement with the literature supports within‐tendon signals to potentially serve as an IBF indicator.

## 3 Discussion

The Achilles is the largest and most clinically significant tendon, due to its importance in motion and high susceptibility to injury,^[^ ^3^ ^]^ where an increase in rupture injuries has been observed over the last 30 years.^[^ ^26^ ^]^ Stemming from modern‐day work‐life, chronic injuries in tendons such as the wrist flexors are also coming into focus.^[^ ^27^ ^]^ With growing awareness of tendinopathy impacting quality of life, there still exists a knowledge gap in the tendon healing mechanism, highlighting the need for more advanced imaging methods. Here, using the non‐invasive MSOT technique, tendon structures, and surrounding vasculature were simultaneously visualized, facilitating morphological characterization of individual fibers and the quantification of oxygenation levels. Real‐time motion dynamics were tracked in action, and perfusion studies showed post‐exercise physiological changes detectable as early as 1.5 min. The increase of vessel length, reported to be a functional measure of Achilles perfusion during exercise,^[^ ^28^ ^]^ was quantified, while increasing oxygenation levels in posterior and superficial vessels were observed.

Overall, the 4D (real‐time volumetric) approach offered by MSOT constitutes a significant advancement for tendon imaging upon current methods: MRI; due to its poor temporal resolution, and US; where the typically 2D approach limits accuracy and reproducibility. Diagnosis of tendon ruptures and splinters are some potential uses of clinical MSOT. Another potentially fitting application is the monitoring after repair surgeries. For example, FDL and FHL are commonly used as graft sources for a deficient TP (e.g., adult flatfoot deformity),^[^ ^29^ ^]^ and cadaver studies have thus been used to test post‐reconstruction effects where flexion force was decreased. MSOT could be a viable option for motion assessment postreconstruction. In addition, visualization of graft integration in vivo including the vasculature response can be of great value.

Unlike contrast‐enhanced ultrasound or MRI, MSOT has the capacity to simultaneously image tendons and surrounding vasculature in a label‐free manner. The method can further contribute to advancing our understanding of tendon healing. The role blood supplies play in such a process is still unclear, with some considering IBF as a marker of tendinopathy,^[^ ^5^ ^]^ while contradicting reports suggest IBF increase to be a normal physiological response to exercise. The Achilles perfusion study unveiled diverse hemodynamic responses post‐exercise across different vasculature groups surrounding the tendon, which is a key step toward understanding the physiology underlying regeneration. Furthermore, MSOT sensitivity has been indicated to be superior to that of Doppler US, where changes in IBF were detected at 1.5 min after exercise, in contrast to at least 30 min reported for the latter modality.^[^ ^7^, ^27^, ^30^ ^]^

Additionally, MSOT could provide feedback during rehabilitation. There is increasing evidence that spotlights the important role patient psychology plays during the recovery period.^[^ ^31^ ^]^ After injuries such as Achilles tendon rupture, patients could undergo months of physiotherapy and treatment, during which time motivational levels drop due to the slow functional improvement. Recently, it has been shown that increasing patient motivation during rehabilitation leads to the reduction of kinesiophobia, and positively affects recovery outcome.^[^ ^32^ ^]^ MSOT's sensitivity could provide visualization of tendon neovascularisation, a precursor in the tendon healing process, and this positive feedback on the effectiveness of the treatment protocol could encourage patients and increase rehabilitation incentives. In the future, MSOT could even drive the development of patient‐specific treatments for the alleviation of chronic pain.

The current study was limited to healthy subjects where statistical significance of the perfusion study is further impaired by the low sample size. With this successful pilot demonstration, a dedicated follow‐up study with a larger pool of samples would yield more clinically relevant quantifications. From the imaging performance perspective, if the characterization of faster tendon dynamics would be desired, higher repetition frequency lasers can be used to enable faster imaging rates.^[^ ^33^ ^]^ Hardware developments to increase the ultrasound detection bandwidth could further improve the spatial resolution and penetration depth, while super‐resolution optoacoustic imaging has recently been demonstrated.^[^ ^34^ ^]^ Another potential shortcoming is the absence of skin type impact exploration, typically critical for optoacoustic‐based approaches, as all volunteers imaged fall within Type I to Type III on the Fitzpatrick scale. An extenuating fact is tendons are small and shallow structures relative to other clinical MSOT applications (e.g., carotid), potentially alleviating the issue. Overall, a thorough investigation with wide diversity demographics encompassing all skin types would be needed to assess the actual application‐specific impact of skin type, which would be the first step toward developing a solution addressing this critical issue.

## 4 Conclusion

In conclusion, the first MSOT imaging study toward noninvasive functional evaluation of human tendons was performed, including the wrist flexors, ankle, and Achilles tendons. Unique in its ability to offer real‐time, volumetric, label‐free imaging with molecular contrast, and compounded by the method's relatively low cost, MSOT has been shown capable of assessing dynamic tendon morphology and hemodynamic physiology in response to controlled motion and exercise. Larger studies with increased cohort diversity would lead to more robust clinically relevant metrics, thus empowering the technique as a new clinical imaging tool for routine tendon examinations.

## 5 Experimental Section

### MSOT Imaging System

**Figure 5:**
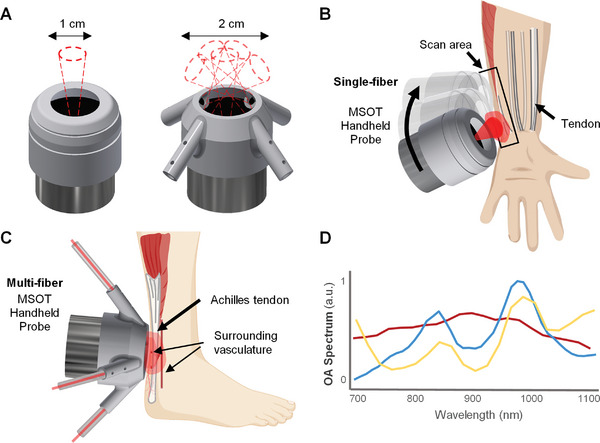
Experimental procedure for human tendon in vivo imaging with multispectral optoacoustic tomography (MSOT). A) 3D design models of single‐fiber (left) and multi‐fiber (right) holders for the handheld probe, depicting the illuminated field‐of‐views. B) Illustration of large‐volume scanning using the single‐fiber probe, where an extended region‐of‐interests (black rectangle) is scanned. C) The multi‐fiber probe configuration for static multispectral quantitative imaging of the Achilles tendon. D) Normalized MSOT absorption spectrum of in vivo artery (red), in vivo tendon (yellow), and ex vivo sheep tendon (blue).

The handheld MSOT imaging probe consists of a custom‐made spherical matrix US array (Imasonic SaS, Voray, France) composed of 256 piezo composite elements, which allows for the collection of volumetric tomographic data covering a volume of ≈1.5 × 1.5 × 1.5 cm^3^, with an approximately isotropic spatial resolution of ≈200 µm. The laser source employed was an optical parametric oscillator‐based laser (Innolas, GmbH, Krailling, Germany), tuneable between 680 and 1250 nm, and generates short <10 ns pulses at 10 Hz pulse repetition rate. The laser fluence at the tissue surface was below 20 mJ cm^−^^2^ at all the illumination wavelengths, thus conforming to the ANSI laser safety limits for human skin exposure.^[^ ^35^ ^]^ The excitation light was delivered through fiber bundles with either a single or a 5‐arm output, enabled via separate 3D‐printed probe‐holder designs depicted in **Figure** **5**, panel a. The single‐output configuration provides more narrowly focused illumination and was used to produce large‐volume reconstructions where a large‐area scan sequence was stitched using a Fourier‐based spatial compounding (stitching) algorithm,^[^ ^36^ ^]^ as illustrated in Figure 5b. Alternatively, the multi‐fiber bundle was employed for static‐probe applications (Figure 5c) including real‐time dynamic imaging and multispectral sequences, as the more outspread fluence pattern achieves a larger effective field‐of‐view (>2 cm along the lateral *xy* plane, Figure S4, Supporting Information). Finally, probe encapsulation by agar and the further use of US gel ensures acoustic coupling. MSOT signals were sampled in parallel using a custom‐built 40 Msps data acquisition system (DAQ, Falkenstein Microsystems GmbH, Taufkirchen, Germany). A custom graphic user interface (GUI) software providing live preview^[^ ^37^ ^]^ was used during the acquisition, offering real‐time visualization and feedback to ensure optimal probe positioning. An imaging frame rate of 10 Hz was achieved, limited by the laser repetition rate. Images were reconstructed using back‐projection,^[^ ^38^ ^]^ and a linear spectral unmixing algorithm was used to resolve the bio‐distribution of oxygenated (HbO) and deoxygenated (HbR) hemoglobin components.^[^ ^39^ ^]^ All image reconstruction, processing, and ANOVA analyses were carried out using MATLAB (Version 9.1, R106, MathWorks Inc, Massachusetts, USA). Quantification measurements were also done in ImageJ (NIH, Bethesda, Maryland, USA), as well as Amira (Zuse Institute Berlin, Germany).

### Tendon Absorption Spectra Imaging Protocol

As tendons consist mainly of type I collagen, its strong absorption spectrum in the near‐infrared (NIR) optical window was highly advantageous for in vivo deep‐tissue MSOT imaging. Absorption spectrum analysis of hemoglobin and collagen bio‐distributions was performed between 700 and 1100 nm in 20 nm steps on an ex vivo sheep Achilles tendon (obtained from a local slaughterhouse) as well as in vivo human wrist tendon and artery (Figure 5d). The collagen spectra exhibited characteristic absorption peaks that could be exploited for identification. Also taking into consideration of the hemoglobin spectrum for the optimization of image quality and signal‐to‐noise ratio, single‐wavelength tendon scans were henceforth carried out at 880 nm.

### Healthy Volunteers

In this work, eight healthy (4F, 4m) volunteers were recruited and tendons from the wrist (flexor carpi ulnaris, flexor carpi radialis, and the palmaris longus), ankle (flexor digitorium longus, FDL; flexor hallicus longus, FHL; and tibialis posterior, TP), and heel (Achilles) were imaged in vivo. This noninterventional preliminary study does not fall within the scope of the Human Research Act (HRA) as considered by the Zurich Cantonal Ethics Commission, thus no authorization from the ethics committee was required. Informed consent was obtained from each healthy volunteer. Age and gender were all noted for each volunteer and included in the hemodynamic results, as these two factors are known to have effects on the perfusion of the Achilles.^[^ ^40^ ^]^

### Ethics Approval

This noninterventional preliminary study does not fall within the scope of the Human Research Act (HRA) as considered by the Zurich Cantonal Ethics Commission, thus no authorization from the ethics committee was required.

## Conflict of Interest

The authors declare no conflict of interest.

## Supporting information

Supporting Information

Supplemental Video 1

Supplemental Video 2

## Data Availability

The data that support the findings of this study are available from the corresponding author upon reasonable request.
