# Supplementary material for: Multispectral Optoacoustic Tomography Enables In Vivo Anatomical and Functional Assessment of Human Tendons
Source: Adv Sci (Weinh). 2024 Mar 6;11(18):2308336. doi: 10.1002/advs.202308336 (PMC11095142; doi:10.1002/advs.202308336)
Supplement: Supplementary file 1 — Supporting Information [file ADVS-11-2308336-s003.pdf]

ADVANCED SCIENCE
Open Access

Supporting Information

for *Adv. Sci.*, DOI 10.1002/advs.202308336

Multispectral Optoacoustic Tomography Enables In Vivo Anatomical and Functional Assessment of Human Tendons

*Ivana Ivankovic, Hsiao-Chun Amy Lin, Ali Özbek, Ana Orive, Xosé Luís Deán-Ben and Daniel Razansky**

Supporting Information

Multispectral Optoacoustic Tomography Enables *in vivo* Anatomical and Functional Assessment of Human Tendons

Ivana Ivankovic¶, Hsiao-Chun Amy Lin¶, Ali Özbek, Ana Orive, Xosé Luís Deán-Ben, and Daniel Razansky*

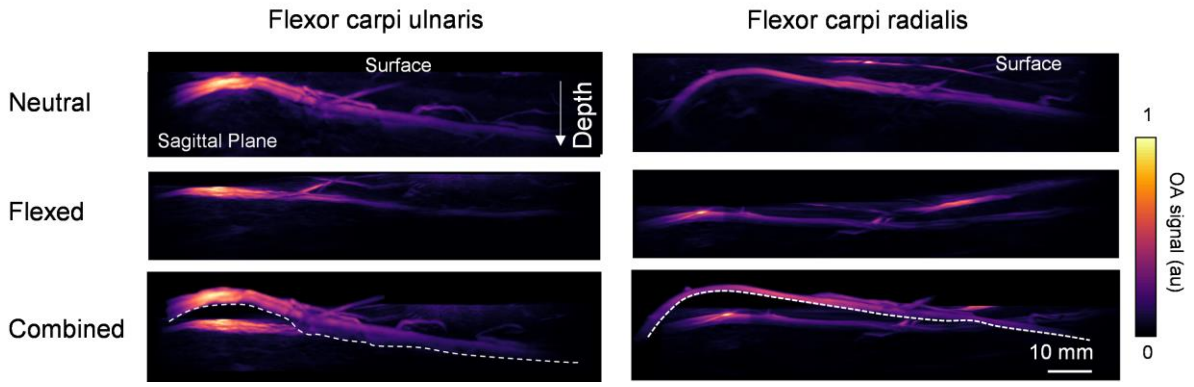

Figure S1: Spatially compounded scans of the flexor carpi ulnaris and flexor carpi radialis in the sagittal plane in neutral and flexed states. Combined images of the neutral and flexed states show differences in anatomy of tendon.

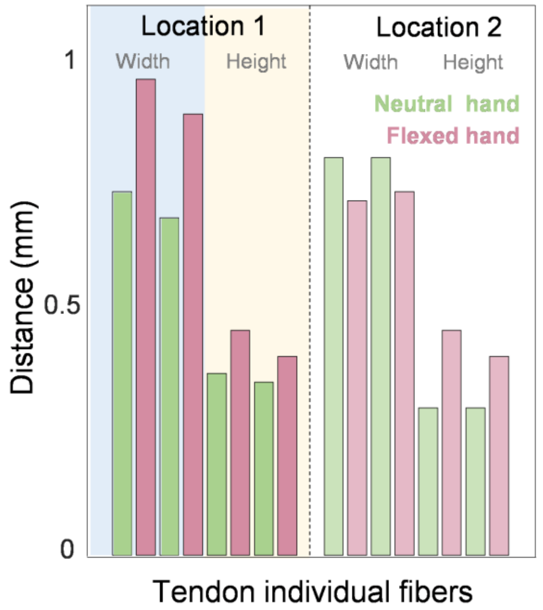

Figure S2: Measurements (mm) of width (blue shade) and height (orange shade) of individual fibers in the Palmaris Longus tendon for two locations in two hand positions, opened hand (green) and closed hand (pink).

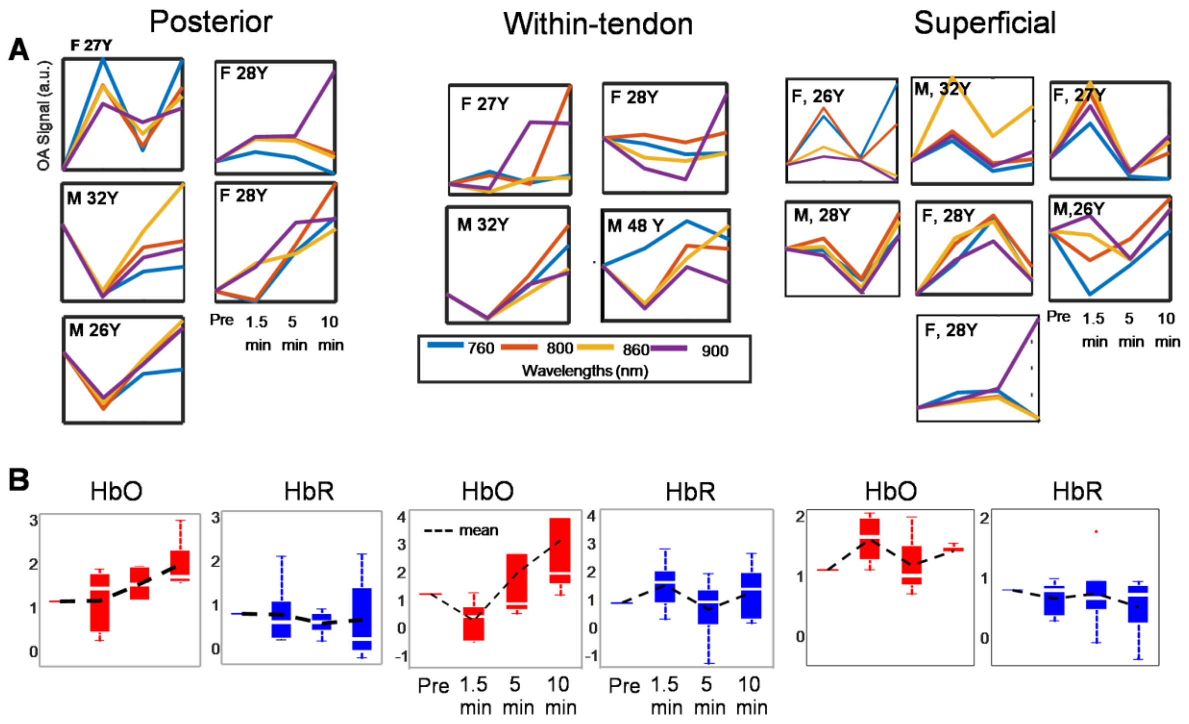

Figure S3: Multispectral analysis of perfusion dynamics for three distinct groups of vasculature: posterior, within, and superficial to tendon. **a.** OA signal intensity for 760, 800, 860 and 900 wavelengths per volunteer for four different time points: pre-exercise and 1.5, 5, and 10 minutes post exercise. **b**. Group unmixing results for oxygenated (oxy) and deoxygenated (deoxy) hemoglobin for all time points.

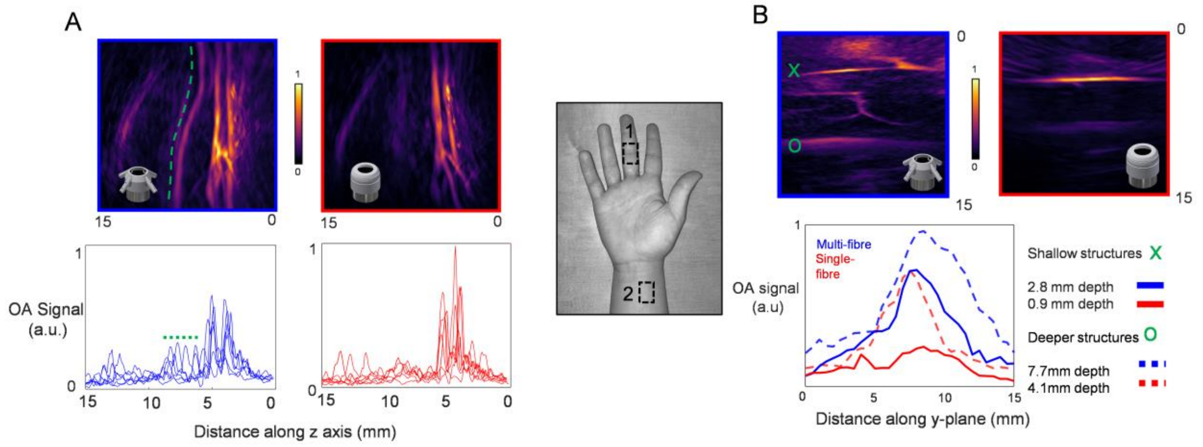

Figure S4: *In-vivo* characterization of multi-fiber (blue) and single-fiber (red) holders. **a.** Represents OA signal intensity in depth (z axis) characterization of the middle finger, where green represents features visible from multifiber use. **b.** Represents OA signal intensity in y-plane, where signal is higher and more homogenous for the multifiber.

Supporting Information: (Video)

Video S1: MSOT scan of the wrist flexor tendon carpi ulnaris. The continuous frame sequence was compounded via a Fourier-based spatial algorithm to stitch together a large-volume reconstruction, revealing the complete elongated structure spanning from the wrist towards the middle of the forearm. An artery could be seen pulsating at around frame 190.

Video S2: Sagittal (*xy*) and coronal (*xz*) MSOT projections of the ankle during flexion-extension motion, capturing real-time dynamics of the flexor hallicus longus (FHL), flexor digitorium longus (FDL), and tibialis posterior (TP) tendons.
